# Identification and ranking of recurrent neo-epitopes in cancer

**DOI:** 10.1186/s12920-019-0611-7

**Published:** 2019-11-27

**Authors:** Eric Blanc, Manuel Holtgrewe, Arunraj Dhamodaran, Clemens Messerschmidt, Gerald Willimsky, Thomas Blankenstein, Dieter Beule

**Affiliations:** 1 Core Unit Bioinformatics, Berlin Institute of Health, Charitéplatz 1, Berlin, 10117 Germany; 2 Institute of Immunology, Charité - Universitätsmedizin Berlin, corporate member of Freie Universität Berlin, Humboldt-Universität zu Berlin, and Berlin Institute of Health, Lindenberger Weg 80, Berlin, 13125 Germany; 3 Max Delbrück Center for Molecular Medicine in the Helmholtz Association (MDC), Robert-Rössle-Str. 10, Berlin, 13092 Germany; 4 Berlin Institute of Health, Charitéplatz 1, Berlin, 10117 Germany; 5 Charité - Universitätsmedizin Berlin, corporate member of Freie Universität Berlin, Humboldt-Universität zu Berlin, and Berlin Institute of Health, Charitéplatz 1, Berlin, 10117 Germany; 6 German Cancer Research Center (DKFZ), Im Neuenheimer Feld 280, Heidelberg, 69120 Germany

**Keywords:** Cancer, Immunotherapy, Neo-epitope, Neo-antigen, Precision treatment

## Abstract

**Background:**

Immune escape is one of the hallmarks of cancer and several new treatment approaches attempt to modulate and restore the immune system’s capability to target cancer cells. At the heart of the immune recognition process lies antigen presentation from somatic mutations. These neo-epitopes are emerging as attractive targets for cancer immunotherapy and new strategies for rapid identification of relevant candidates have become a priority.

**Methods:**

We carefully screen TCGA data sets for recurrent somatic amino acid exchanges and apply MHC class I binding predictions.

**Results:**

We propose a method for *in silico* selection and prioritization of candidates which have a high potential for neo-antigen generation and are likely to appear in multiple patients. While the percentage of patients carrying a specific neo-epitope and HLA-type combination is relatively small, the sheer number of new patients leads to surprisingly high reoccurence numbers. We identify 769 epitopes which are expected to occur in 77629 patients per year.

**Conclusion:**

While our candidate list will definitely contain false positives, the results provide an objective order for wet-lab testing of reusable neo-epitopes. Thus recurrent neo-epitopes may be suitable to supplement existing personalized T cell treatment approaches with precision treatment options.

## Background

Increasing evidence suggests that clinical efficacy of cancer immunotherapy is driven by T cell reactivity against neo-antigens [1–5]. While not yet fully understood, immune response and recognition of tumor cells containing specific peptides depends critically on the ability of the MHC class I complexes to bind to the peptide in order to present it to a T cell. Neo-antigens can be created by a multitude of processes like aberrant expression of genes normally restricted to immuno-privileged tissues, viral etiology or by tumor specific DNA alterations that result in the formation of novel protein sequences. Furthermore there is now evidence for neo-epitopes generated from alternative splicing [6] and alterations in non-coding regions [7].

With the advent of affordable short read sequencing, comprehensive neo-antigen screening based on whole exome sequencing has become feasible and many cancer immune therapeutic approaches try to utilize detailed understanding of the neo-epitope spectrum to create additional or boost pre-existing T cell reactivity for therapeutic purposes [8, 9]. However, in practice the selection and validation of the most promising neo-epitope candidates is a difficult and time-consuming task. The typical approach is based on the private mutational catalogue of the individual patient: exome sequencing data is subjected to bioinformatics analysis and used to predict neo-epitopes and their binding affinities to the MHC class I complex. Our study aims to complement this approach by a precision medicine perspective. We search and prioritize neo-epitope candidates which have a high potential for neo-antigen generation and are likely to appear in multiple patients. These neo-antigens hold the potential for development of *off the shelf T cell therapies* for sub groups of cancer patients. We use epidemiological data to give rough estimates for the expected number of patients in these groups.

Candidate prediction always relies on somatic variant detection workflows and affinity prediction algorithms based on machine learning, see e.g. [10]. Binding prediction far from perfect [11] especially for rarer HLA types, and may also depend on mutational context [12]. Catalogues of the neo-epitope landscape across various cancer entities have been created by various authors [13–15]. While neoantigen landscape is diverse and sparse [13], here we provide an unbiased, comprehensive ranking of candidates, defined as neo-epitopes arising from recurrent mutations, predicted to be binding to a specific HLA-1 allele. The candidates are ranked according to the expected number of target patients.

## Methods

### Data sets

**Fig. 1:**
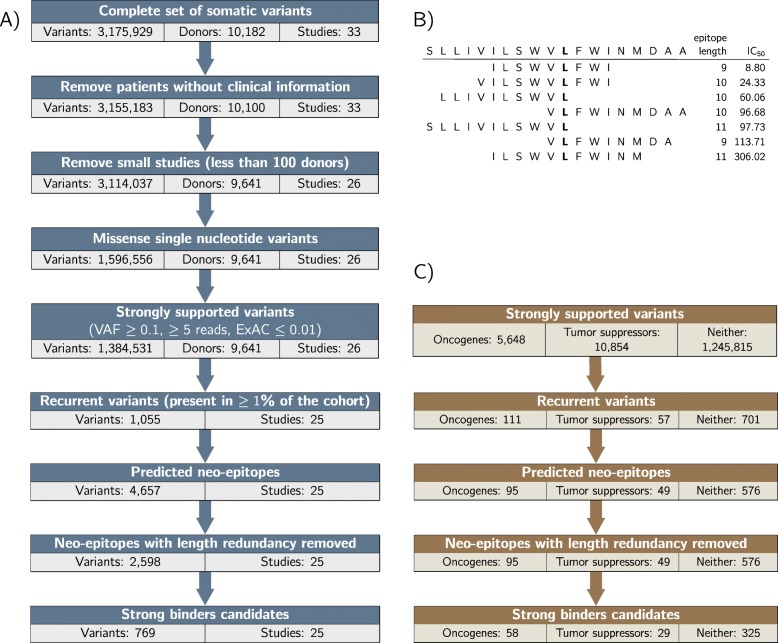
Workflow overview **a** Overview of the recurrent neo-epitope candidates generation process: TCGA studies are selected for at least 100 donors with clinical annotations. For each of these studies, recurrent strongly supported missense Single-Nucleotide Variants are collected. Neo-epitopes binding to 11 HLA-1 types are predicted, redundancy is removed from that set (see B) and strong binders are retained. **b** Example of epitope redundancy: the 18 amino-acids long sequence surrounding recurrent variant GLRA3:S274L generates 7 binding neo-epitopes for the type HLA-A*02:01. Our pipeline retains only the strongest predicted binder for a given variant and HLA-1 type pair (the first, with an IC_50_ of 8.8 nM in the example). **c** Number of SNVs occuring in genes classified as Oncogenes or Tumor Suppressors by Vogelstein et al. [28], at various point of the variant selection and neo-epitope selection process

Somatic variants for different cancer entities have been determined using matched pairs of tumor and blood whole exome or whole genome sequencing in the TCGA consortium. We downloaded the open-access somatic variants from GDC data release 7.0 [16], consisting of 33 TCGA projects and 10,182 donors in total. Details of the somatic variant calling can be found in [17]. We excluded patients without corresponding entries in the clinical information tables, and 7 projects with less than 100 samples, yielding 9,641 samples covering 26 cancer studies. Figure 1 provides an overview of the complete bioinformatics process, from the GDC somatic single nucleotide variants to the identification of the candidates.

### Variant selection

For each sample we selected all single nucleotide variants obtained by the “mutect2” pipeline, that had a “Variant_Type” equal to “SNP”, a valid ENSEMBL transcript ID and a valid protein mutation in “HGVSp_Short”. From these variants, we selected those with a “Variant_Classification” equal to “Missense_Mutation”. We checked that all variants had a “Mutation_Status” equal to (up to capitalisation) “Somatic”, that the total depth “t_depth” was the sum of the reference “t_ref_count” and the alternate “t_alt_count” alleles counts, and that the genomics variant length is one nucleotide. To avoid high number of false positives we consider only variants that are supported by at least 5 reads and have a VAF of at least 10%. Furthermore we removed any variant that occurs with more than 1% in any population contained in the ExAC database version 0.31 [18], by coordinates liftover from the GRCh38 to hg19 human genome versions. This way we obtained 26 cancer entity data sets containing a total of 9,641 samples with an overall 1,384,531 variants.

### Recurrent protein variant selection

We define recurrence strictly on the protein/amino acid exchange level, i.e. different nucleotide acid variants leading to the same amino acid exchange due to code redundancy will be counted together. Recurrent protein variants are defined within each TCGA study. A protein variant is deemed recurrent when it appears in at least 1% of all the patients in the cohort. As cancer types are only considered when the number of patients involved in the studies is greater than 100, this threshold ensures that every recurrent variant has been observed in at least 2 patients for a given cancer type. To be conservative, the recurrence frequency has been computed using, for the denominator, all patients with clinical information in the study, including those without high-confidence missense SNVs. Using this definition, the total number of recurrent amino acid changes is 1055. A variant recurrent in multiple cancer types is counted multiple times in the above number, the number of unique recurrent variants regardless of the cancer is 869. Additional file 1 shows the most frequent amino acid exchanges across 25 cancer entities, as no variant from project TCGA-KIRC’s donors is labeled as recurrent.

Recurrent variants occurring at the same positions (for example when gene’s IDH1 codon R132 is mutated to amino acid H, C, G or S) have been merged into 819 variants suitable for comparisons with the cancer hot spots lists [14]. 122 out of the 819 merged variants belong to the set of 470 cancer hotspot variants, and 5 (PCBP1:L100, SPTLC3:R97, EEF1A1:T432, BCLAF1:E163 & TTN:S3271) to the set of presumptive false positives hotspots listed in the supplementary material of [14].

### MHC class i binding prediction and epitopes selection

For all recurrent variants identified, we assess *in silico* their predicted propensity that the amino-acid exchange generates a binding neo-epitope.

A variety of machine learning algorithms have been developed to determine the MHC binding *in silico*, see ref. [19] for review. Most methods are trained on Immune Epitope Database (IEDB) [20] entries and use allele specific predictors for frequent alleles, while pan-methods are applied to extrapolate to less common alleles. We predicted the MHC class I binding using NetMHCcons [21] v1.1, which predicts peptides IC_50_ binding, and classifies these predictions as non-binder, weak and strong binders, based on the relative ranking of binding predictions. As the range of IC_50_ binding values strongly depend on the HLA-1 allele [22], we have used the NetMHCcons classification to select our neo-epitope candidates.

For a given recurrent variant and a given HLA-1 type, the epitope prediction pipeline can produce multiple overlapping epitopes candidates, differing by their length and/or their position (see Fig. 1B). To remove such size redundancy, only the epitope with the lowest predicted mutant sequence IC_50_ is retained. This procedure also removes non-overlapping epitopes, to keep only at most one epitope per recurrent protein variant and HLA-1 type. For comparison we also compute the IC_50_ for the respective wild type peptide.

For MHC class I binding prediction we selected 11 frequent HLA-1 types: HLA-A*01:01, HLA-A*02:01, HLA-A*03:01, HLA-A*11:01, HLA-B*07:02, HLA-B*08:01, HLA-B*15:01, HLA-C*04:01, HLA-C*06:02, HLA-C*07:01, HLA-C*07:02. We limited the search for poly-peptides 9, 10 and 11 amino-acids long. For these alleles, we obtain 769 strong binding recurrent peptides and 1829 weak binders, over all considered cancer types. Their complete list is in Additional file 2, where each candidate is listed with the HLA-1 type it is preticted to bind to.

### Data QC

To ensure that the proportion of variants caused by technical artifacts is small, we have computed the proportion of SNVs called in poly-A, poly-C, poly-G or poly-T repeats of length greater than 6 have been computed for each data study [23], for unique variants (that occur in only one patient across a project cohort), and for variants that are observed more than once in a cohort (Additional file 3). For comparison, we have computed the expected frequency of such events, assuming that all possible 11-mers (the mutated nucleotide at the center, flanked by 5 nucleotides on each side) are equiprobable, regardless of their sequence.

Based on this equiprobable model, we have computed the probability that the number of mutations found in repeat locii is equal to or greater than the observed numbers. When considering variants appearing more than once, this probability is not significant for all studies; when unique variants are considered, those appear in repeat locii significantly more often than expected by chance in 7 out of 26 studies (TCGA-COAD, TCGA-KIRP, TCGA-LIHC, TCGA-READ, TCGA-SKCM, TCGA-TGCT & TCGA-UCAC, significance level set to 0.05 after Benjamini-Hochberg multiple testing correction).

### Mice

ABabDII mice (described in detail in [24]) have been used for this study. They are transgenic for entire human *TCR*- *α* and *TCR*- *β* gene loci, as well as for *HHD* molecule [25] and deficient for the murine *Tcr*- *α* and - *β* chains, as well as for murine *β2m* and *H2*- *D*^*b*^ genes. The mice used in the study were generated and housed under SPF conditions (caged enriched with bedding material, 3-5 mice/cage, standard light/dark cycle, food and water ad libitum) at the Max-Delbrück-Center animal facility. All animal experiments were approved by the Landesamt für Arbeitsschutz, Gesundheitsschutz und technische Sicherheit, Berlin, Germany.

### Generation of mutation-specific t cells in ABabDII mice

For each candidate, 3 ABabDII mice between 8 to 12 weeks old (6 in total) underwent immunisation. They were injected subcutaneously with 100 *μ*g of mutant short peptide (9-10mers, JPT) supplemented with 50 *μ*g CpG 1826 (TIB Molbiol), emulsified in incomplete Freund’s adjuvant (Sigma). Repetitive immunizations were performed with the same mixture at least three weeks apart. Mutation-specific CD8 ^+^ T cells in the peripheral blood of immunized animals were assessed by intracellular cytokine staining (ICS) for IFN *γ* 7 days after each boost. All 6 animals were peptide-reactive. The 6 mice were sacrificed for spleen preparation by cervical dislocation after isofluran anasthesia.

### Patient number estimates and HLA-1 frequencies

HLA-1 frequency data *f*_*h*_ for the U.S. population was retrieved from the Allele Frequency Net Database (AFND) [26]. Frequency data were estimated by averaging the allele frequencies of multiple population datasets from the North American (NAM) geographical region. The major U.S. ethnic groups were included and sampled under the NAM category. Cancer incidence data for the U.S. population (*N*_*d*_) was retrieved from the GLOBOCAN 2012 project of the International Agency for Research on Cancer, WHO [27].

Assuming that the fraction of a recurrent variant in the U.S. population affected by cancer entity *d* (*r*_*d*_) is identical to the observed ratio of that variant in the corresponding TCGA study, the number of patients of HLA-1 type *h* whose tumor contain the variant is expected to be

$$ n_{h} = f_{h} \sum_{d} r_{d} N_{d}. $$

The summation runs over 18 diseases *d* for which both the TCGA projects and the cancer incidence data are available.

## Results

### Recurrent variants and candidates

From the GDC repository [16], we have collected somatic variants for 33 TGCA studies. After removing patients without clinical meta-data, and studies with less than 100 patients, we have selected 1,384,531 high-confidence missense SNPs from 9,641 patients, see methods for details. Using this data, 1,055 variants are deemed recurrent (Additional file 1), as they can be found in more than 1% of the patients in the respective study cohort. These recurrent variants correspond to 869 unique protein changes, as some appear in multiple cancer entities. 77 of the recurrent variants occur in at least 3% of their cohort (43 unique protein changes).

From these 869 unique protein changes, we have generated candidates that are predicted to be strong MHC class I binders in frequent HLA-1 types that we considered for initial selection. 415 (48%) of them lead to a strong binder prediction. In total, there are 772 candidates that are recurrent in a cancer entity cohort, and predicted as binding for a considered HLA-1 type. These candidates are non-redundant among all the 9-, 10- & 11-mers containing the variant: the selection process retains only the peptide sequence with the lowest predicted IC_50_. Figure 1 and Table 1 provide an overview of the variant selection and neo-epitope candidates generation processes, while Additional file 2 lists all neo-epitopes (weak and strong predicted binders) after removing redundancy.

**Table 1:** Overview of the 33 TCGA studies used in this analysis

Project name	Number of patients		Variants per patients		Missense variant per patient		Recurrent variants	Strong binders
	Total	With clinical data	Average	Median	Average	Median		
TCGA-BLCA	412	412	326	226	157	109	22	14
TCGA-BRCA	986	986	123	62	50	25	8	10
TCGA-CESC	289	289	358	157	143	62	17	16
TCGA-COAD	399	397	666	176	288	82	41	34
TCGA-ESCA	184	184	246	187	95	73	80	72
TCGA-GBM	393	390	212	70	93	36	15	5
TCGA-HNSC	508	508	201	139	97	66	14	3
TCGA-KIRC	336	336	79	69	33	31	0	0
TCGA-KIRP	281	281	85	82	39	38	5	2
TCGA-LAML	143	143	69	15	16	6	14	7
TCGA-LGG	508	507	70	36	33	16	14	0
TCGA-LIHC	364	364	149	120	70	58	11	16
TCGA-LUAD	567	515	367	242	180	113	7	0
TCGA-LUSC	492	492	368	301	187	153	20	19
TCGA-OV	436	435	173	121	58	47	10	7
TCGA-PAAD	178	178	168	50	77	19	24	12
TCGA-PCPG	179	179	13	12	5	4	8	5
TCGA-PRAD	495	495	59	35	27	15	3	7
TCGA-READ	137	136	475	148	232	70	320	186
TCGA-SARC	237	237	119	70	45	26	2	0
TCGA-SKCM	467	467	841	472	413	229	266	220
TCGA-STAD	437	437	488	157	211	74	17	14
TCGA-TGCT	144	128	23	21	9	8	9	6
TCGA-THCA	492	492	22	12	6	5	4	3
TCGA-THYM	123	123	39	24	10	4	6	2
TCGA-UCEC	530	530	1672	149	708	54	118	109
TCGA-ACC	92	92	117	36	0	0	0	0
TCGA-CHOL	51	45	110	62	0	0	0	0
TCGA-DLBC	37	37	173	157	0	0	0	0
TCGA-KICH	66	66	44	25	0	0	0	0
TCGA-MESO	82	82	47	44	0	0	0	0
TCGA-UCS	57	57	183	67	0	0	0	0
TCGA-UVM	80	80	23	16	0	0	0	0
Total	10182	10100	Total number: 3155183		Total number: 1384531		1055	769

The 7 studies displayed at the bottom have not been used for the determination of recurrent vairants, as the number of patients is less than 100. The number of strong binders includes all occurrences of neo-epitopes candidates, so a candidate may be counted multiple times when it is predicted to be binding several HLA-1 types

Despite large differences between variant selection protocols, 123 variants deemed recurrent by the above process can be found among the 470 variants identified in the cancer hotspot datasets [14] (Additional file 4). This overlap is strongly dependent on how frequent those variants are observed: there are 54 common variants out of the 61 variants observed more than 10 times over our dataset (>88%). Among the 819 variants retained for the comparison (see methods for details), only 5 appear among the variants flagged as possible false positive by Chang et al. (<1%).

### Enrichment in known cancer related genes

We observe that recurrent variants occur substantially more frequently in known cancer-related genes than in other genes (Fig. 1c). Initially approximatively one percent of all observed variants are found in genes that have been described [28, 29] as oncogenes (54 genes) or tumor suppressor genes (71 genes). When recurrent unique protein changes are considered, the fraction of known oncogenes or tumor suppressor genes is substantially increased to 13% and 6.5% respectively (a *χ*^2^ test between unique protein changes and unique recurrent variants gives a *P* value smaller than 10^−16^). These fractions only marginally increase to 14% and 7% when only the unique protein changes leading to predicted strong binders for frequent HLA-1 types are considered (a *χ*^2^ test between unique recurrent variants and strong binders gives a non-significant *P* value). Additional file 5 shows a similar enrichment of known cancer-related genes per cohort. We observe that the enrichment is stronger for oncogenes than for tumor suppressors. This might be expected, as activating mutations in oncogenes are mainly distributed on a few protein positions, while loss of function mutations in tumor suppressors are generally distributed more broadly along the protein sequence.

It is interesting to observe that several of the highly prevalent neo-epitope candidates occur in genes that are involved in known immune escape mechanisms: RAC1:P29S is recurrent in study SKCM (melanoma), is predicted to lead to strong binding neo-epitopes for HLA-A*01:01 and HLA-A*02:01, and is reported to up-regulate PD-L1 in melanoma [30]. CTNNB1:S33C is recurrent in studies LIHC (liver hepatocellular carcinoma) and UCEC (uterine corpus endometrial carcinoma), is predicted to lead to strong binding neo-epitopes for HLA-A*02:01, and has been shown to increase the expression of the Wnt-signalling pathway in hepatocellular carcinoma [31], leading to modulation of the immune response [32] and ultimately to tumor immune escape [33]. In a separate study, Cho et al. [34] show that this mutation confers acquired resistance to the drug imatinib in metastatic melanoma. Finally, FLT3:D835Y recurrent in study LAML (acute myeloid leukemia), is predicted to lead to a strong binding neo-epitope for HLA-A*01:01, HLA-A*02:01 and HLA-C*06:02, and following Reiter et al. [35], Tyrosine Kinase Inhibitors promote the surface expression of the mutated FLT3, enhancing FLT3-directed immunotherapy options, as its surface expression is negatively correlated with proliferation.

While the described mechanisms are probably sufficient to explain immune escape in tumor evolution, the candidates could nevertheless be viable targets for adoptive T cell therapy or TCR gene therapy.

### Recurrent neo-epitopes in patient populations

Upon assumption of statistical independence, the product of the frequency of a recurrent variant with the frequency of class I alleles in the population and the incidence rates of cancer types provides an estimate for the number of patients that carry that specific candidate. Using the number of newly diagnosed patients per year and HLA-1 frequency in the US population, we are able to compute the expected number of patients for 18 cancer entities for which both cancer census data and a TCGA study are available. The occurrence numbers for individual candidates range from 0 to 2,254 for PIK3CA:H1047R in breast cancer patients of type HLA-C*07:01; Table 2 presents a summary of expected patient numbers for the complete set of candidates. We estimate that, in the US alone, the previously discussed RAC1:P29S mutation might be present in 628 new patients carrying the HLA-A*02:01 allele each year (in 556 melanoma patients and in 72 lung small cell, head & neck or uterine carcinomas patients, see Additional file 6 for details). For the CTNNB1:S33C mutation, the total number of HLA-A*02:01 patients in the US is expected to be 364, from uterine corpus, prostate and liver cancer types. As another example, 115 myeloid leukemia patients in the US are expected to be of type HLA-A*02:01 and carry the FLT3:D835Y mutation.

**Table 2:** Expected number of newly diagnosed U.S. patients by HLA-1 type and cancer entity

A)													
Cancer entity	Study	Number of patients	HLA-A*01:01 (7.61%)	HLA-A*02:01 (20.36%)	HLA-A*03:01 (6.60%)	HLA-A*11:01 (4.37%)	HLA-B*07:02 (6.51%)	HLA-B*08:01 (4.80%)	HLA-B*15:01 (4.46%)	HLA-C*04:01 (16.69%)	HLA-C*06:02 (5.72%)	HLA-C*07:01 (9.28%)	HLA-C*07:02 (15.39%)
Bladder Urothelial Carcinoma	BLCA	69300	340	638	197	152	142	112	89	223	376	596	383
Invasive Breast Carcinoma	BRCA	204800	110	1090	461	199	108	375	120	482	1411	2305	1234
Cervical Squamous Cell Carcinoma	CESC	14000	58	135	16	17	13	14	23	142	27	66	152
Colon Adenocarcinoma	COAD	154840	861	1628	770	760	450	222	375	2961	1019	1620	1800
Esophageal Adenocarcinoma	ESCA	4750	23	151	26	16	20	5	13	101	23	26	83
Glioblastoma Multiforme	GBM	3204	7	17	3	7	0	0	3	8	5	3	3
Head & Neck Squamous Cell Carcinoma	HNSC	58000	43	208	112	45	81	11	81	301	136	220	175
Renal Clear Cell Carcinoma	KIRC	57600	13	70	23	0	0	8	0	0	10	0	26
Papilliary Renal Cell Carcinoma	KIRP	8064	0	6	0	3	0	0	0	29	0	0	0
Acute Myeloid Leukemia	LAML	13500	77	115	0	8	49	0	0	0	48	9	73
Hepatocellular Carcinoma	LIHC	29700	12	496	131	84	69	12	7	68	69	112	174
Lung Squamous Cell Carcinoma	LUSC	66000	181	642	328	168	282	13	77	593	151	172	366
Serous Ovarian Cancer	OV	16800	26	24	33	10	15	2	14	39	9	14	36
Prostate Adenocarcinoma	PRAD	260000	120	852	69	69	34	50	70	175	387	628	1202
Melanoma	SKCM	75000	2649	7890	1817	936	861	530	203	2186	438	1000	2457
Stomach Adenocarcinoma	STAD	25000	47	172	56	66	26	8	30	206	114	166	130
Thyroid Cancer	THCA	46400	394	0	0	0	0	14	0	0	0	0	44
Endometrial Carcinoma	UCEC	55000	942	2804	817	501	369	290	493	2222	856	1602	1779
Total		1161958	5904	16936	4858	3033	2517	1666	1598	9736	5080	8539	10116
B)													
Number of candidates in diseases			55	91	68	64	33	24	24	48	48	50	55

A) Expected number of patients of a given HLA-1 type who harbor at least one potentially immunogenic neo-epitope candidate for that HLA-1 type. Both the cancer incidence and the allele frequency are estimated for the U.S. population. The probability that a patient carries at least one variant from the set of neo-epitope candidates is computed under the assumption that the occurrence of variants in a cancer patient stems from statistically independent events. B) Number of neo-epitope candidates identified in the 18 studies shown in A, which are predicted to be strong binders to the corresponding HLA-1 type

**Fig. 2:**
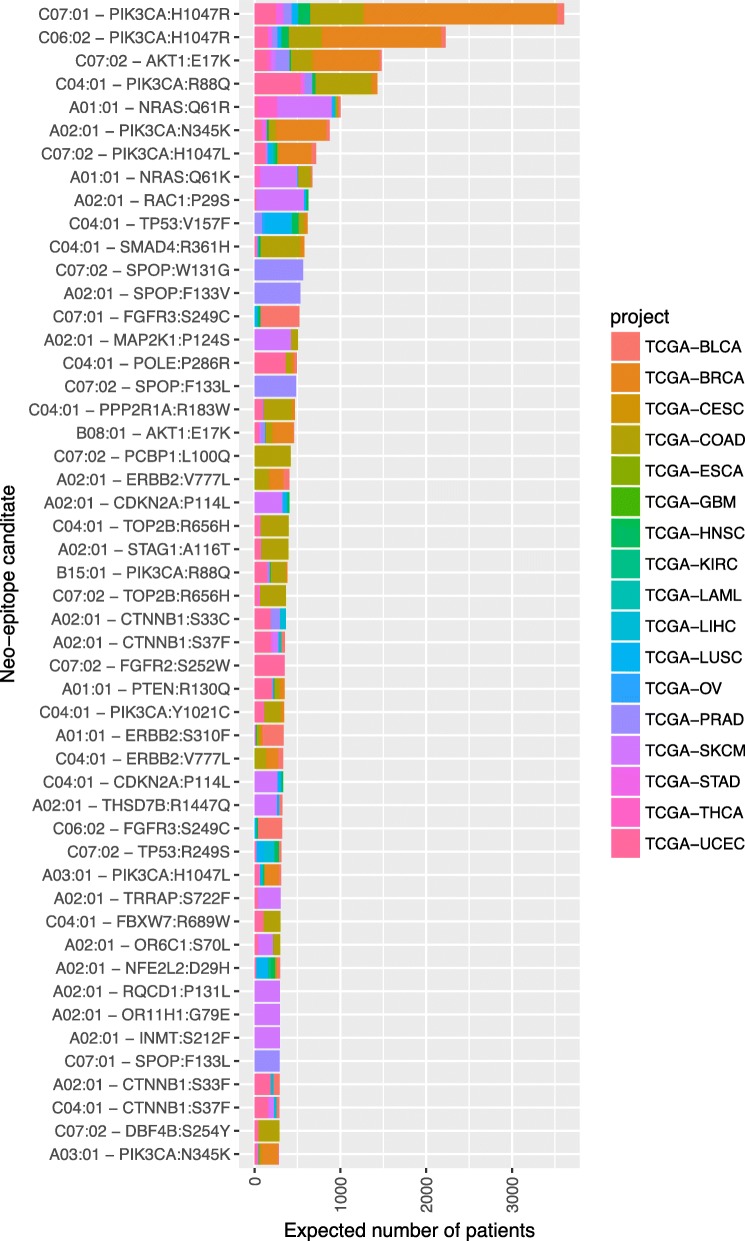
50 most frequent candidates in patients for which strong MHC I binding is predicted. For each candidate, the expected number of patients is obtained by summing over the 18 cancer entities for which the number of newly diagnosed patients in the US is available, and for which a corresponding TCGA study has been included in our analysis

Figure 2 shows the cumulative expected number of patients that carry a specific epitope, and with matching HLA-1 type, for the 50 candidates with the highest expected patients number. The number of patients is derived from the sum over all cancer entities, including those in which the candidate is not recurrent according to our criteria. For example, among newly diagnosed US patients of type HLA-C*04:01, 88 prostate cancer patients are expected to carry the mutation PIK3CA:R88Q, even though its observed frequency in the PRAD study is as low as 0.2%. The data shown in Fig. 2 can be found in Additional file 6.

### Accessible patient population

**Fig. 3:**
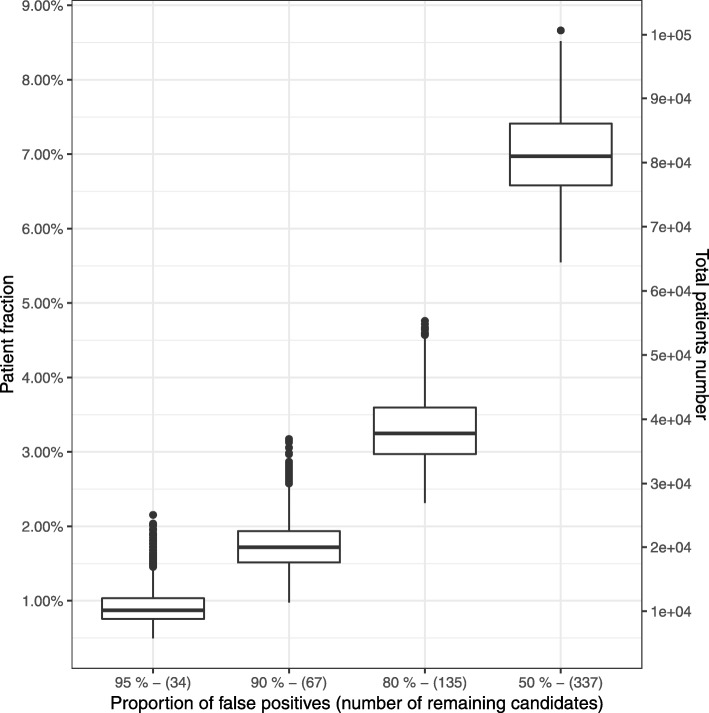
Expected influence of the proportion of false positive neo-epitope candidates on the patient population. Proportion of the patients that carry at least one neo-epitope candidate mutation, and whose HLA-I allele set contains the candidate HLA type, when a limited percentage of the neo-epitope candidates is considered. The patient cohort considered here consists of 6868 patients from the 18 TCGA cohorts for whom the HLA types are known. For each false positive proportion, the false positive candidates have been selected 1000 times at random

As our current understanding of peptide immunogenicity is still incomplete [36], not all candidates predicted by our pipeline can be expected to trigger an immunogenic response in patients. To further evaluate the usefulness of our results we consider the list of candidates (neoepitope and HLA type pairs) selected form our ranking. Assuming a T cell therapy could be generated for every candidate we can compute the number of patients that would benefit, see methods. Because of imperfections in candidate prediction, not all candidates hold the potential for an effective T cell therapy, and these ineffective candidates can be thus viewed as “false positives”. Because it is impossible to create a reliable estimated for the fraction of these false positives due to the complexity of the underlying algorithm and biological process we decided to consider a broad range of possible values from 50% to 95%, cf. Figure 3. Using a subset of 6868 patients for whom HLA types were known, we predict the number of patients for whom such positive response might be expected, as a function of the proportion of “false positives” in our candidates. To estimate the impact of such “false positives”, we have randomly flagged 1000 times 337, 539, 607 & 640 candidates as “false positives”, which is corresponding to a fraction of about 50%, 80%, 90% and 95% of the total 674 candidates. This procedure left us with 1000 sets of 337, 135, 67 & 34 candidates that were not flagged as “false positives”. Figure 3 shows that for a pessimistic 90% of false positive candidates, more than 1.5% of patients over all cancer entities (95% CI between 1.25% & 2.65%, mean 1.78%, median 1.72%, both corresponding to about 20000 new patients per year in the U.S.) are still expected to carry at least one of the 67 remaining candidates’ mutation and corresponding HLA allele. While the proportions are modest, the absolute number of patients seems relevant. The figure in Additional file 7 shows that there are considerable differences between entities: the proportion of matching patients is much higher in diseases with high mutational load such as melanomas (TCGA-SKCM, median about 9% for 90% false positives), than in diseases with lower mutational load, such as thyroid cancer (TCGA-THCA, 0.2%, 90% false positives).

### Confirmational evidence

A limited validation of our method was performed in two steps: first, we confirmed that our pipeline was able to identify candidates that have been previously reported as eliciting spontaneous CD8 ^+^ T-cell responses in cancer patients in whom the target epitopes were subsequently discovered [37, 38]. Both sets together (Additional file 8) contain 37 epitopes, 35 of which could be mapped to an ENSEMBL transcript (33 unique genes). For 27 of these epitopes our pipeline predicted strong binding with the specific HLA-1 type reported in the corresponding wet-lab investigations. Another 5 epitopes where predicted as weak binders, some of the latter are also predicted to be strong binders in other HLA-1 types. Our pipeline classified 70% of a set of known tumor neo-antigens as strong binders and another 14% as weak binders.

4 out of 34 unique identifiable variants studied by van Buuren et al. [38] and Fritsch [37] are found among our set of high confidence missense variants, but only one (CTNNB1:S37F) fulfills the 1% recurrence threshold (9 uterine carcinoma patients). This variant was shown to trigger immunological response against HLA-A*24:02 [39], which isn’t in the set of alleles that we have systematically tested. However, our prediction show that the same peptide might also be reactive against HLA-C07:02.

Finally, the CDK4:R24C peptide (sequence ACDPHSGHFV, see Additional file 8) is not predicted to bind to HLA-A*02:01, even though it leads to confirmed T cell response [40], and has been related to cutaneous malignant melanoma and hereditary cutaneous melanoma [41], [42]. Taken together, these results show that our candidate prediction pipeline is able to recapitulate most clinically validated neo-epitopes reported in [38] and [37], and that some of these neo-epitopes occur from recurrent variants.

**Fig. 4:**
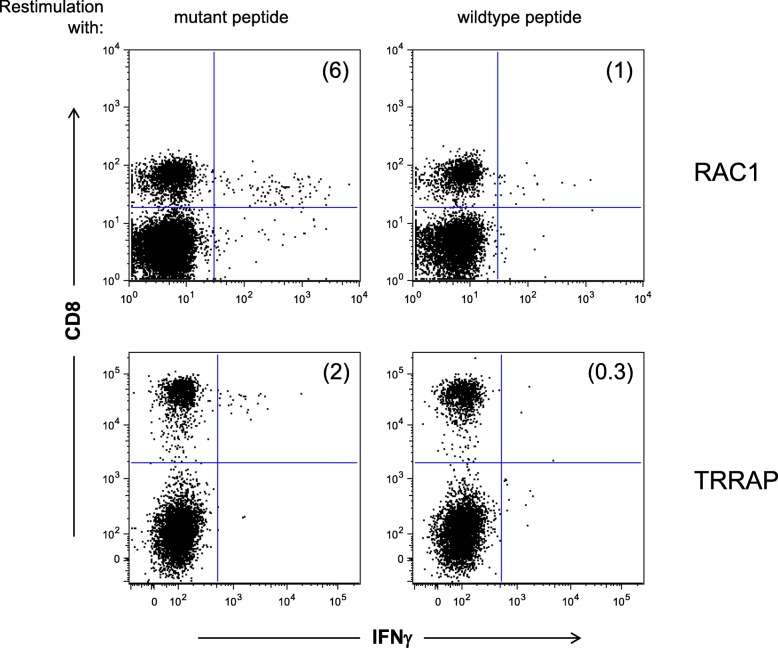
Recognition of predicted epitopes by CD8 ^+^ T cells. Epitopes for recurrent mutations that have been identified *in silico* to bind to HLA-A*02:01 using our pipeline were synthesized and used for immunization of human TCR transgenic ABabDII mice. Examples (RAC1:P29S and TRRAP:S722F) of *ex vivo* ICS analysis of mutant peptide immunized ABabDII mice 7 days after the last immunization are shown. Polyclonal stimulation with CD3/CD28 dynabeads was used as positive control, stimulation with an irrelevant peptide served as negative control (data not shown)

We have also performed preliminary validation for two candidates: RAC1:P29S & TRRAP:S722F binding to HLA-A*02:01 (Fig. 4). We utilized ABabDII mice, transgenic animals that harbour the human TCR *αβ* gene loci, a chimeric HLA-A2 gene and are deficient for mouse TCR *αβ* and mouse MHC I genes. These mice have been shown to express a diverse human TCR repertoire [24, 43] and thus mimic human T cell response. They were immunized at least twice with mutant peptides and IFN *γ* producing CD8 ^+^ T cells were monitored in *ex vivo* ICS analysis 7 days after the last immunization. CD8 ^+^ T cells were purified from spleen cell cultures of reactive mice using either IFN *γ*-capture or tetramer-guided FACSort. Sequencing of specific TCR *α* and *β* chain amplicons that were obtained by RACE-PCR revealed that this procedure yields an almost monoclonal CD8 ^+^ T cell population (not shown). In both cases, tested neo-antigen candidates lead to T cell reactivity, confirming not only predicted MHC binding by our pipeline but also immunogenicity in vivo in human TCR transgenic mice. Therefore this workflow also allows to generate potentially therapeutic relevant TCRs to be used in the clinics for cancer immunotherapy.

## Discussion

By virtue of the underlying mutational processes, the genome architecture and accessibility as well as for functional reasons within the disease process, certain somatic mutations will be present in multiple patients while still being highly specific to the tumor [14]. Using existing cancer studies and neo-epitope binding predictions to MHC class I proteins, we propose a ranking of candidates which mutation occur frequently in observed cancer patient cohorts. The candidates are ranked according to the expected number of target patients. For one candidate, the target patients are defined as those who bear the candidate’s mutation, and whose HLA types contain the candidate’s. The expected number of target patients is proportional to the HLA type frequency in the population, and to the frequency of the mutation in the cancer cohorts. Taking into account the fact that MHC binding is a necessary but not sufficient condition for T cell activity, and the limitations of MHC binding prediction algorithms, our method provides an objective ranking of neo-epitopes based on recurrent variants, as a basis for the development of off-the-shelf immunotherapy treatments.

Despite numerous mechanisms of immune evasion, neo-epitopes are important targets of endogenous immunity [5]. In some cases at least, it has been shown that they contribute to tumor recognition [44], achieve high objective response (in melanoma, see ref. [45, 46]), and a single of them is presumably sufficient for tumor regression [47]. Moreover, positive association has been shown between antigen load and cytolytic activity [48], activated T cells [13] and high levels of the PD-1 ligand [49]. Taken together, these results suggest that neo-epitopes occupy a central role in regulating immune response to cancer, and that this role can be exploited for cancer immunotherapy. Even though the question of negative selection for strong binding neo-epitopes and its relation to other immune evasion mechanisms like HLA loss or PD-L1, CTLA4 dis-regulation is still open [50]. A recent CRISPR screen suggest that more then 500 genes are essential for cancer immunotherapy [51].

Targeting neo-epitopes based on non-recurrent, *private* somatic variants requires generation of private TCRs or CARs for each individual patient, which is challenging [52]. Successful treatments based on genetically engineered lymphocytes has been shown for epitopes arising from unmutated proteins, i.e. *public epitopes*: MART-1 and gp100 proteins have been targeted in melanoma cases [53]. In another trial, Robbins et al. [54] have studied long-term follow-up of patients who were treated with TCR-transduced T cells against NY-ESO-1, a protein whose expression is normally restricted to testis, but which is frequently aberrantly expressed in tumor cells. They show that treatment may be effective for some patients. These results show that immune treatments based on *public* variants can be beneficial, suggesting that similar success may potentially be achieved using candidates based on recurrent variants.

However, targeting such non somatic epitopes presents safety and efficacy concerns [2]. The administration of T cells transduced with MART-1 specific T-cell receptor have led to fatal outcomes [55]. Cross-reactivity of TCR against MAGE-A3 (a protein normally restricted to testis and placenta) caused cardiovascular toxicity [56]. Neo-epitopes based on recurrent somatic variants potentially alleviate such problems, as the target sequences are truly restricted to tumor cells.

Our computation of expected targetable patient groups assumes that neither the cancer type nor the patient’s mutanome are associated with the patient’s HLA-1 alleles. In a recent study, Van den Eyden et al. [50] show that there is little (if any) antigen depletion due to the negative selection pressure from the immune response. Molecular evolution methods applied to somatic mutations show that nearly all mutations escape negative selection [57]. Taken together, these results suggest that the expected probability of a recurrent variant being present in a patient somatic mutations pool should not be affected (significantly) by the patient’s HLA-1 alleles.

The neo-epitope landscape is diverse and sparse [13]. Few neo-epitopes are predicted to be both strong binders and present in multiple patients. In their analysis, Hartmaier et al. [58] estimate that neo-epitopes suitable for precision immuno-therapy might be relevant for about 0.3% of the patients, which is in agreement with our results. However, the absolute number of patients is still considerable, see Table 2. Our study shows that a relatively large number of patients (about 1% of newly diagnosed patients) might benefit from a small library of candidates proven to generate immunological response. These numbers must be compared to “conventional” personalised immunotherapy, where a immunologically active candidate must be identified for each new patient for which efficacy and safety are always unknown. Even if a substantial part of the neo-epitopes we suggest turns out to be false positives due to the limitation of prediction algorithms and understanding of immune response, there is potential to help tens of thousands of patients.

## Conclusions

Off the shelf immune treatments can be faster, less costly and safer for individual patients, because each neo-epitope based treatment scheme can be reused on hundreds of patients per year. In this respect, they might open the way to supplement existing personalized cancer immune treatments approaches with precision treatment options.

We believe that our ranking provides a rational order for testing for and selecting off the shelf neo-epitope based therapies. Our preliminary in vivo mouse experiments show that this in principle feasible.

## Supplementary information

**Additional file 1** 1055 recurrent variants identified in 26 TCGA studies. For each variant, the number of cases harboring the variant (Number of occurrences), the cohort size and the fraction of cases in the cohort (Fraction) are given. When available, COSMIC entries (from ENSEMBL) are are also listed, as well as the highest allele frequency from all populations quoted in ExAC version 0.31 ([18]). Gene annotations from Vogelstein et al. ([28]) & Rubio-Perez et al. ([29]) are also provided.

**Additional file 2** Neo-epitope candidates from recurrent variants. Recurrent variants leading to binding (strong and weak binders) neo-epitopes for one of the 11 HLA types considered. Peptide length redundancy has been removed from the variant list, and each variant is listed only once, even if it is recurrent in multiple study cohorts.

**Additional file 3** Frequency of Single Nucleotides Variants (SNVs) that fall in a poly-A, poly-C, poly-G or poly-T sequence of length at least 6. The variants that appear only once in the whole study are colored in blue, while the variants that appear more than once are colored in red. The dotted line shows the expected fraction of such variants, if the sequences were all random. Except for the LIHC study, all variants that occur more than once in the cohort are found in difficult-to-sequence regions less than expected by chance.

**Additional file 4** Overlap between recurrent variants and hotspot variants. The overlap is based on the codon position, so that all variants occurring at the same protein sequence position are pooled together. The recurrent variants that match the alternate codon definition in Chang et al. are added to the overlap. The recurrent variants are pooled by codon and sorted by decreasing occurrence frequency in the study. The overlap between hotspots and highly recurrent variants is high, and the common variants fraction decreases when recurrent variants become less frequent. The overlap between recurrent variants and the list of suspected false positive hotspots compiled by Chang et al. ([14]) is very limited. Inset: Venn diagram of the total overlap between the recurrent variants called in this study, and the hotspot variants described in Chang et al. ([14]).

**Additional file 5** Oncogenes and tumor suppressors. Number of variants occurring in genes classified as tumor suppressor genes and oncongenes by ([28]), for each study. The numbers are given for the full set of variants, among recurrent variants only and among variants leading to neo-epitope candidates. As each protein change is considered only once, the total number of variants is always smaller or equal to the sum over all studies, as protein changes appearing in multiple studies are counted only once in the total.

**Additional file 6** Expected number of target patients for each neo-epitope candidates, for the 18 cancer entities with associated epidemiological data. The expected number of patients is the product between the number of new cases, the observed variant frequency and the HLA type frequency in the US population. The total expected number of patients for each candidate is the sum over the expected number of candidates by study.

**Additional file 7** Expected frequency of patients with at least one candidate not labelled as false positive. For each TCGA cohort, we have selected at random 1000 times 50%, 20%, 10% and 5% from the candidates, to conservately model a high rate of false positive within the candidates. From these selected candidates, we have computed the expected frequency of patients with a HLA-1 allele and a mutation matching at least one selected candidate.

**Additional file 8** Confirmation Status with Gold Standard Data Set. The protein changes described in van Buuren et al. ([38]) and Fritsch et al. ([37]) have been mapped to the ENSEMBL protein set and neo-epitopes have been computed using our standard pipeline. 26 of these epitopes are exactly recovered by the pipeline, for one of them the pipeline predicts a strong binder for a shorter peptide, and 5 of them are predicted to be weakly binding. Column 7 to 10 are copied from ([37]) and ([38]).

**Additional file 9** ARRIVE checklist concerning the animals used for the experimental validation of the in vivo presentation of two peptides.

## Abbreviations

^*a*^: SLC: small cell lung cancer, NSLC: non-small-cell lung cancer
^*b*^: Parent and mutant sequences have been exchanged in Fritsch et al. ([37])
^*c*^: A strong binder is found for the shorter mutant peptide KINKNPKYK
^*d*^: No exact match found by alignment against not redundant human proteins, not found in manual inspection of proteins P19971 and E5KRG5 from gene TYMP
^*e*^: Found by alignment against non-redundant human proteins in EAW69514.1 (melanoma associated antigen (mutated) 1, isoform CRA_e (not in ENSEMBL peptides))
