# Supplementary material for: Identification and ranking of recurrent neo-epitopes in cancer
Source: BMC Med Genomics. 2019 Nov 27;12:171. doi: 10.1186/s12920-019-0611-7 (PMC6882202; doi:10.1186/s12920-019-0611-7)
Supplement: Supplementary file 3 — Additional file 3 Frequency of Single Nucleotides Variants (SNVs) that fall in a poly-A, poly-C, poly-G or poly-T sequence of length at least 6. The variants that appear only once in the whole study are colored in blue, while the variants that appear more than once are colored in red. The dotted line shows the expected fraction of such variants, if the sequences were all random. Except for the LIHC study, all variants that occur more than once in the cohort are found in difficult-to-sequence regions less than expected by chance. [file 12920_2019_611_MOESM3_ESM.pdf]

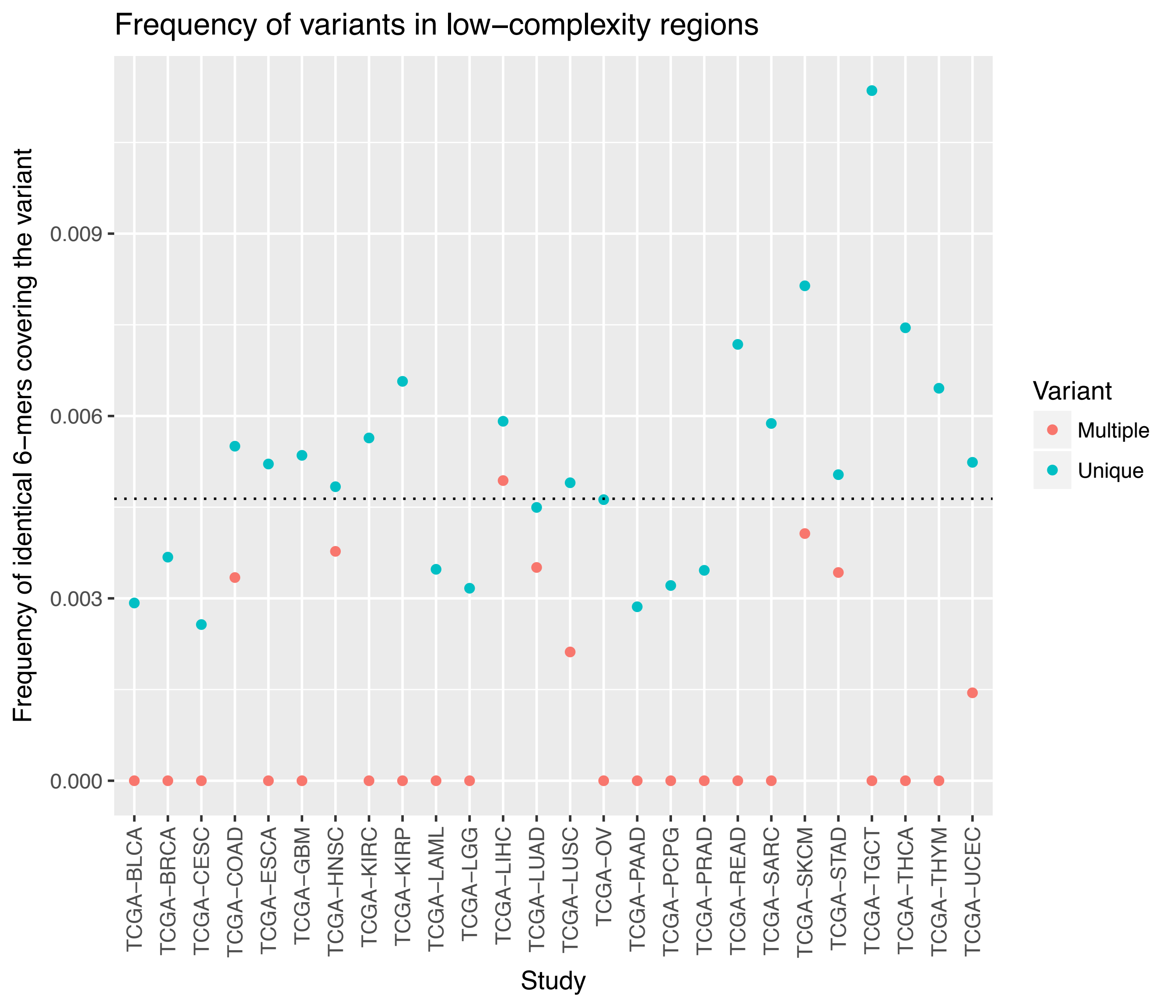

Frequency of variants in low−complexity regions
Frequency of identical 6−mers covering the variant
0.009
0.006
0.003
0.000
Study
TCGA−BLCA
TCGA−BRCA
TCGA−CESC
TCGA−COAD
TCGA−ESCA
TCGA−GBM
TCGA−HNSC
TCGA−KIRC
TCGA−KIRP
TCGA−LAML
TCGA−LGG
TCGA−LIHC
TCGA−LUAD
TCGA−LUSC
TCGA−OV
TCGA−PAAD
TCGA−PCPG
TCGA−PRAD
TCGA−READ
TCGA−SARC
TCGA−SKCM
TCGA−STAD
TCGA−TGCT
TCGA−THCA
TCGA−THYM
TCGA−UCEC
Variant
Multiple
Unique
