# Supplementary figures and images for: Identification and ranking of recurrent neo-epitopes in cancer
Source: BMC Med Genomics. 2019 Nov 27;12:171. doi: 10.1186/s12920-019-0611-7 (PMC6882202; doi:10.1186/s12920-019-0611-7)

# Recurrent variant overlaps with the HotSpot sets

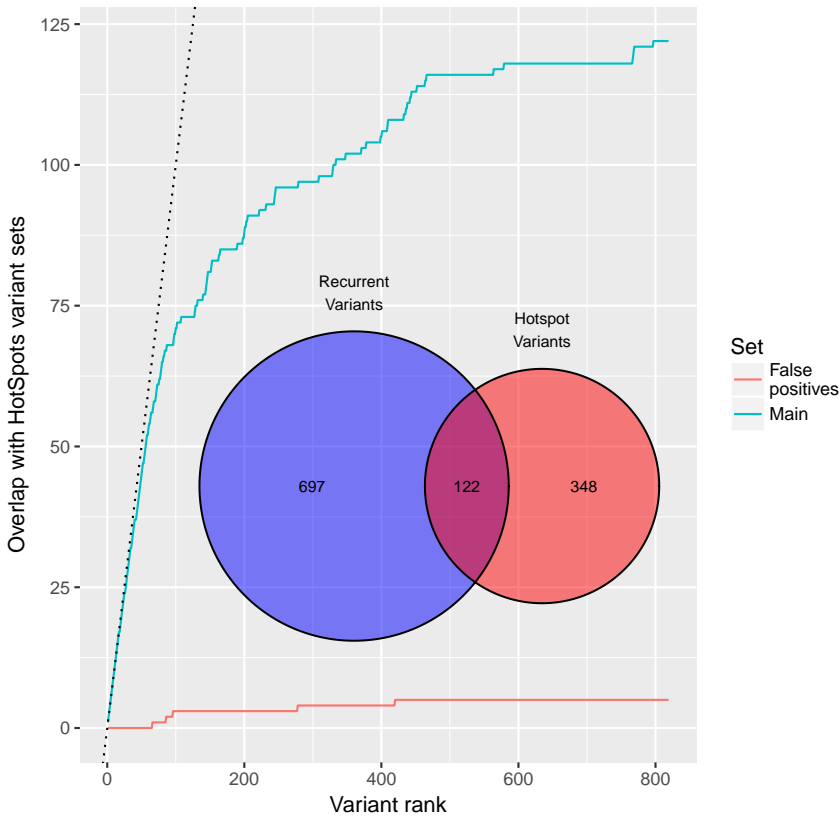

Supplement: Supplementary file 4 — Additional file 4 Overlap between recurrent variants and hotspot variants. The overlap is based on the codon position, so that all variants occurring at the same protein sequence position are pooled together. The recurrent variants that match the alternate codon definition in Chang et al. are added to the overlap. The recurrent variants are pooled by codon and sorted by decreasing occurrence frequency in the study. The overlap between hotspots and highly recurrent variants is high, and the common variants fraction decreases when recurrent variants become less frequent. The overlap between recurrent variants and the list of suspected false positive hotspots compiled by Chang et al. ([14]) is very limited. Inset: Venn diagram of the total overlap between the recurrent variants called in this study, and the hotspot variants described in Chang et al. ([14]). [file 12920_2019_611_MOESM4_ESM.pdf]

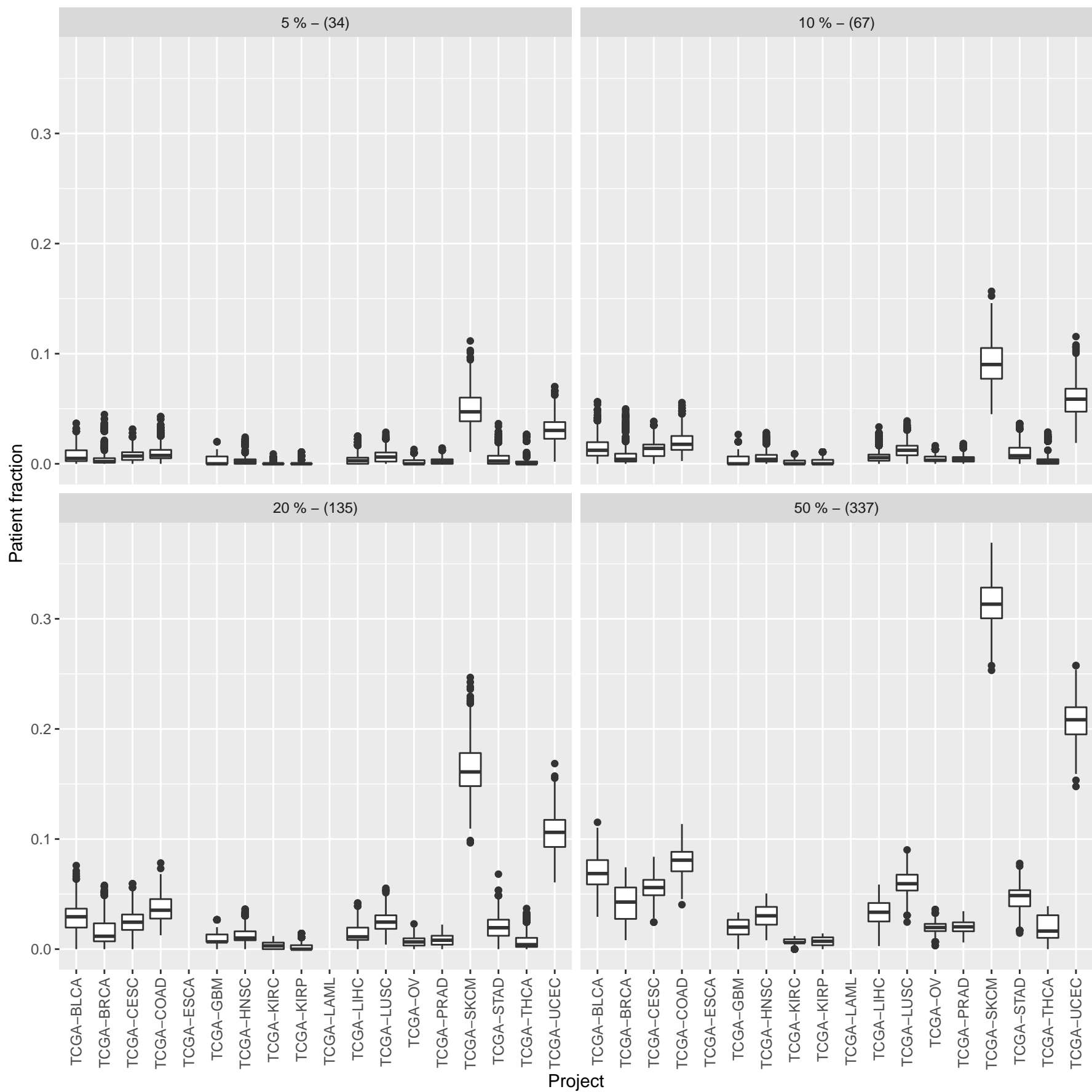

Supplement: Supplementary file 7 — Additional file 7 Expected frequency of patients with at least one candidate not labelled as false positive. For each TCGA cohort, we have selected at random 1000 times 50%, 20%, 10% and 5% from the candidates, to conservately model a high rate of false positive within the candidates. From these selected candidates, we have computed the expected frequency of patients with a HLA-1 allele and a mutation matching at least one selected candidate. [file 12920_2019_611_MOESM7_ESM.pdf]
