# Supplementary material for: Identification and ranking of recurrent neo-epitopes in cancer
Source: BMC Med Genomics. 2019 Nov 27;12:171. doi: 10.1186/s12920-019-0611-7 (PMC6882202; doi:10.1186/s12920-019-0611-7)

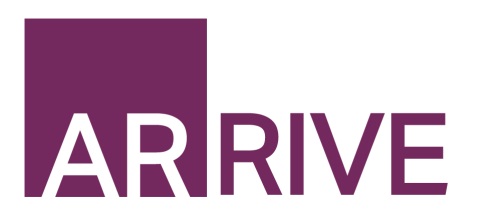


The ARRIVE Guidelines Checklist

Animal Research: Reporting In Vivo Experiments

Carol Kilkenny^1^, William J Browne^2^, Innes C Cuthill^3^, Michael Emerson^4^ and Douglas G Altman^5^

*^1^The National Centre for the Replacement, Refinement and Reduction of Animals in Research, London, UK, ^2^School of Veterinary Science, University of Bristol, Bristol, UK, ^3^School of Biological Sciences, University of Bristol, Bristol, UK, ^4^National Heart and Lung Institute, Imperial College London, UK, ^5^Centre for Statistics in Medicine, University of Oxford, Oxford, UK.*

|  | | ITEM | RECOMMENDATION | Section/ Paragraph |
| --- | --- | --- | --- | --- |
| 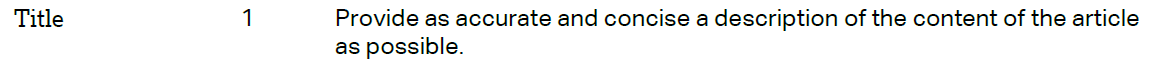 | | | Title |  |
| 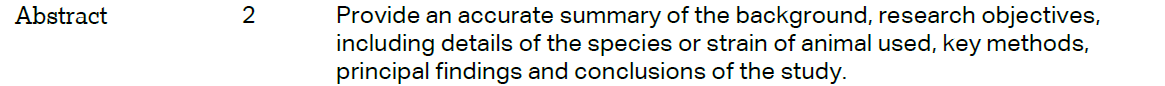 | | | Abstract NA |  |
| INTRODUCTION | | |  |  |
| 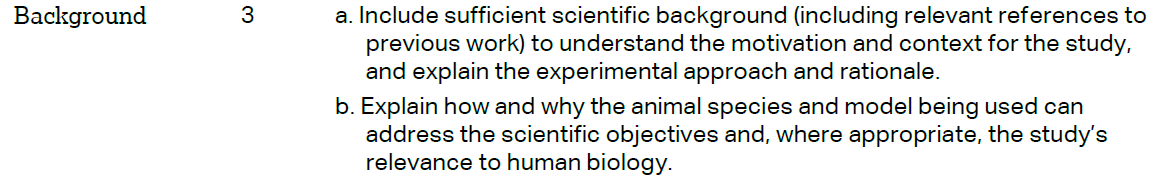 | | | Background Paragraphs 1 & 2 (a)  Results - Confirmational evidence Paragraph 4 (b) |  |
| 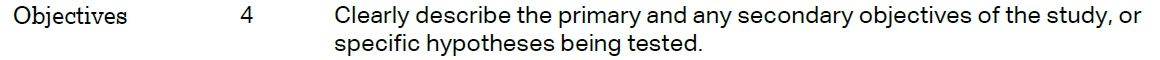 | | | Paragraph 3 |  |
| METHODS | | |  |  |
| 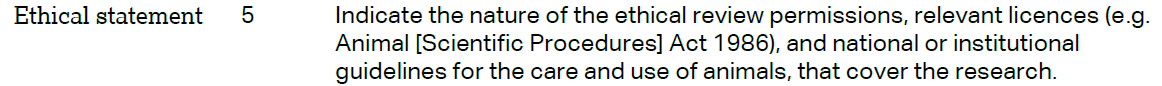 | | | Declaration paragraph 1 |  |
| 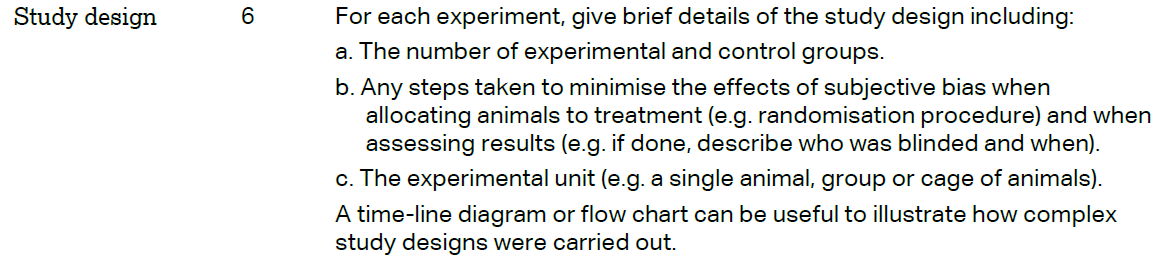 | | | NA |  |
| 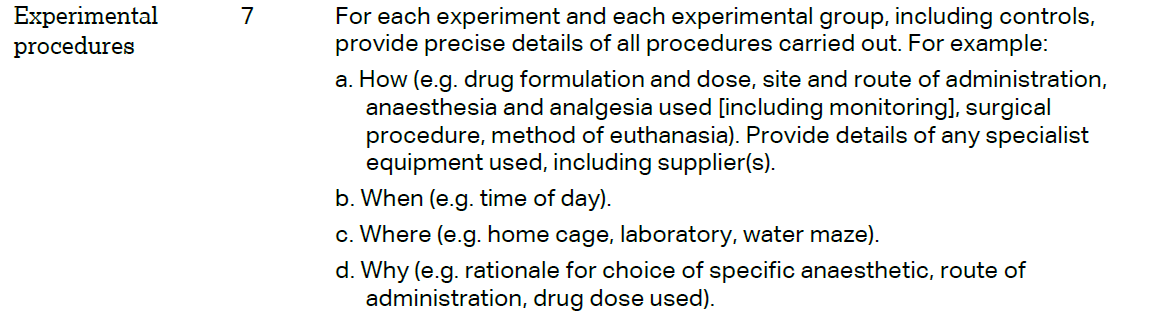 | | | Methods - Mice paragraph 1 (c) &  Methods - Generation of mutation-specific T cells in ABabDII mice paragraph 1 (a)  (b) (c) NA |  |
| 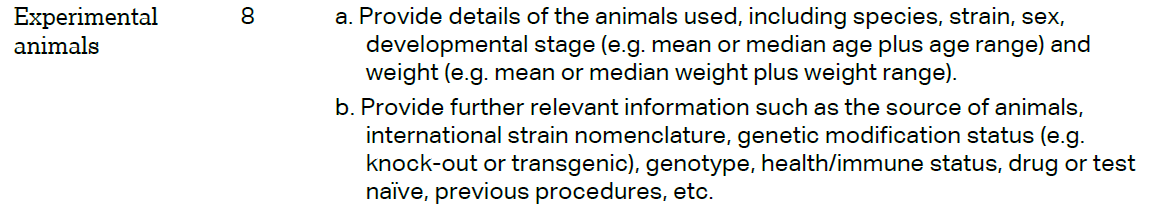 | | | Methods - Generation of mutation-specific T cells in ABabDII mice paragraph 1  (a) (b) |  |

The ARRIVE guidelines. Originally published in *PLoS Biology*, June 2010^1^

| 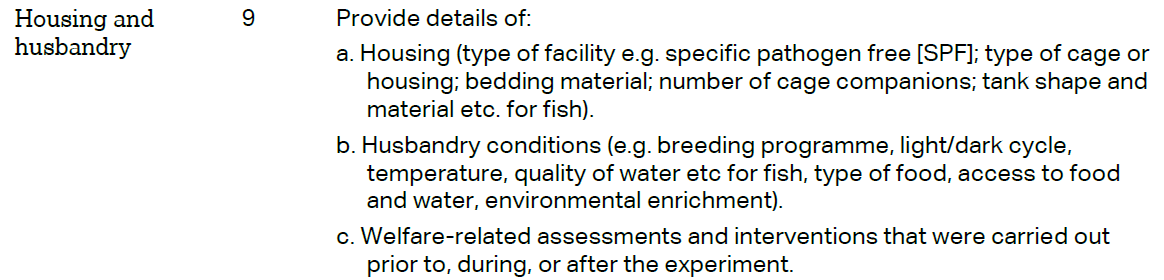 | Methods - Mice paragraph 1  (a) (b)  (c) NA | |
| --- | --- | --- |
| 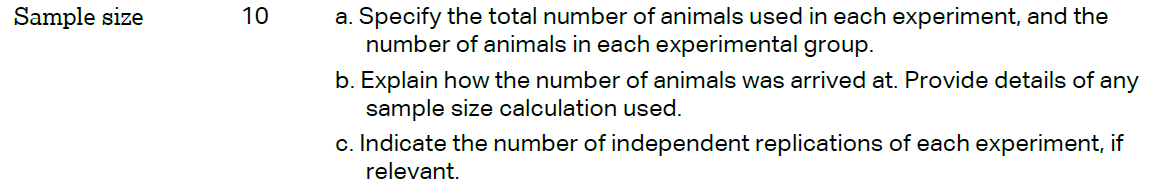 | Methods - Generation of mutation-specific T cells in ABabDII mice paragraph 1  (a) | |
| 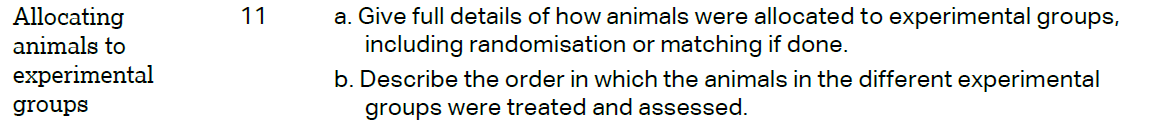 | NA | |
| 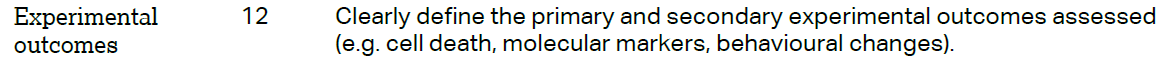 | Results - Confirmational evidence paragraph 4 | |
| 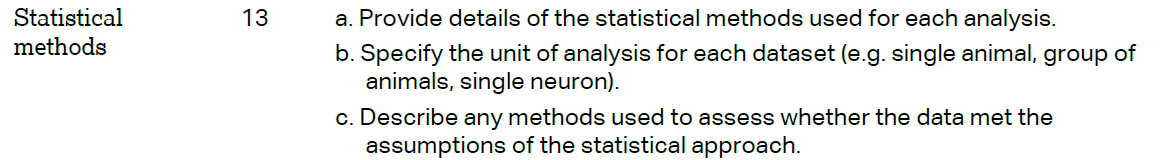 | NA (Sample size as judged from overlapping complete TCR repertoire) | |
| RESULTS |  | |
| 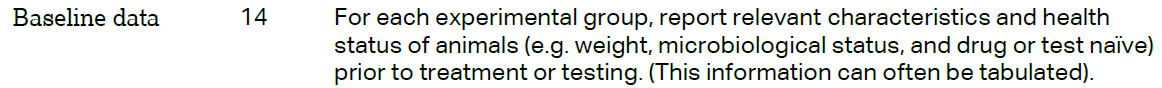 | NA | |
| 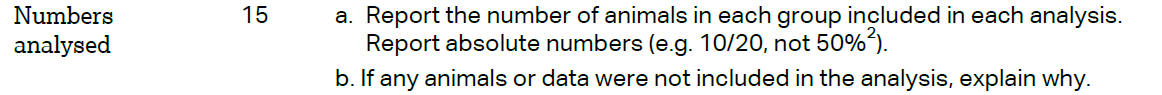 | Methods - Generation of mutation-specific T cells in ABabDII mice paragraph 1 | |
| 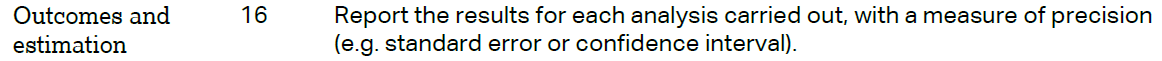 | Results - Confirmational evidence paragraph 4 | |
| 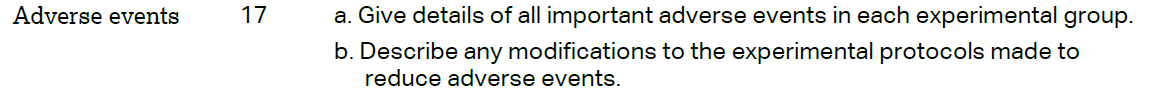 | NA | |
| DISCUSSION |  | |
| 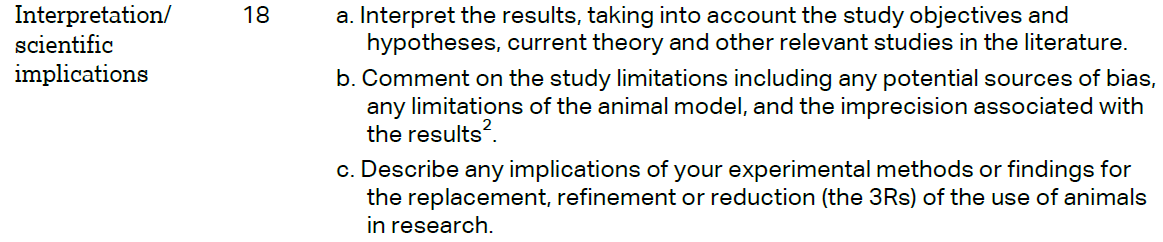 | Discussion paragraph 5 (a)  NA (b)  NA (c) | |
| 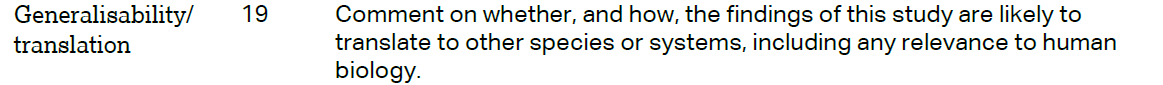 | Results - Confirmational evidence paragraph 4 | |
| 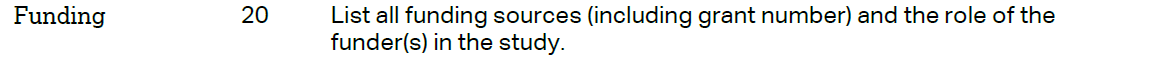 | | Declaration paragraph 5 |


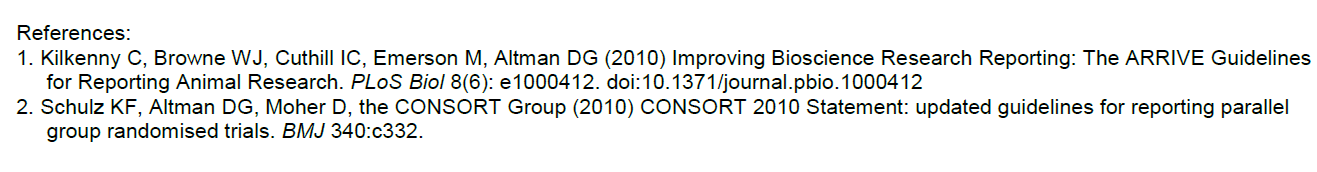

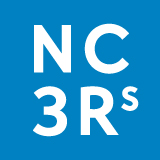

Supplement: Supplementary file 9 — Additional file 9 ARRIVE checklist concerning the animals used for the experimental validation of the in vivo presentation of two peptides. [file 12920_2019_611_MOESM9_ESM.docx]
